# Clozapine-induced cardiac failure

**DOI:** 10.4103/0019-5545.31607

**Published:** 2006

**Authors:** Kuruvilla Thomas, Sr Susan, KK Jayaprakash

**Affiliations:** * Chief Psychiatrist Kusumagiri Mental Health Centre, Kakkanad, Cochin 682030, Kerala, India; ** Psychiatrist Kusumagiri Mental Health Centre, Kakkanad, Cochin 682030, Kerala, India; *** Psychiatrist Kusumagiri Mental Health Centre, Kakkanad, Cochin 682030, Kerala, India

**Keywords:** Dementia, psychotic symptoms, clozapine, cardiac failure, elderly

## Abstract

A 73-year-old woman with dementia was given clozapine for treatment-resistant psychotic symptoms. Subsequently, she developed cardiac failure. Caution should be exercised when using clozapine, especially in the elderly.

## INTRODUCTION

The efficacy of clozapine in treatment-resistant schizophrenia is well documented. It is also useful in the management of psychotic symptoms of dementia but should be used with caution due to its broad side-effect profile.^1^ The prevalence of ECG abnormalities in patients who had been using anti-psychotics other than clozapine was 13.6% which increased significantly to 31.1% after commencement of clozapine treatment.^2^ Adverse cardiac effects such as tachycardia, hypotension and hypertension are the most common,^3^ warranting stoppage in many patients.^4^ Circulatory collapse, arrhythmias, myocarditis, cardiomyopathy, pericarditis and pericardial effusion are also known to occur.^5^ Cardiac failure may develop secondary to myocarditis and cardiomyopathy. In a 10-year naturalistic study, clozapine-treated patients appear to be at risk for death from cardiovascular disease secondary to clozapine-associated medical disorders such as obesity, diabetes, hypertension, and hyperlipidaemia.^6^ Sudden death in the initial phase of treatment due to hypersensitivity myocarditis has been reported.^7^ A case of cardiac failure is described in which the classical features of myocarditis were absent.

## THE CASE

M, a 73-year-old woman, was on treatment for dementia of the Alzheimer type with delusions (DSM-IV 290.12) for the past 3 years. As the psychotic symptoms did not respond to various antipsychotics, she was put on clozapine. Considering her age and the known adverse effects of the drug, a cardiology consultation was done on 13 December 2005. Clinical examination, ECG and echocardiogram were normal. Clozapine was started the next day at a dose of 12.5 mg/day, and gradually increased to 100 mg/day. On 5 January 2006 she developed breathlessness. Clinical examination showed signs of cardiac failure with sinus tachycardia and poor R wave progression in V1–V3. An echocardiogram revealed global left ventricular hypokinesia, mild left ventricular systolic dysfunction, a left ventricular ejection fraction of 40% and moderate mitral regurgitation. Clozapine was stopped. She responded to conventional antifailure measures.

## DISCUSSION

The temporal relationship between the introduction of clozapine and onset of cardiac failure suggests that the drug was responsible. However, she could have had independent age-related myocardial dysfunction. The classical features of myocarditis were absent. The maximum pulse rate in our patient was 92/min whereas in a recent case report^8^ it was 130/min with features such as chest pain and flu-like symptoms—generalized bodyache, nasal congestion, sore throat, fever and eosinophilia. Cardiomegaly is a feature of dilated cardiomyopathy which occurs as a result of myocarditis. However, our patient had no cardiomegaly.

The clinical features of myocarditis are varied. The spectrum includes asymptomatic patients who may have electro-cardiographic abnormalities; patients with signs and symptoms of clinical heart failure and ventricular dilatation; and patients with symptoms of fulminant heart failure and severe left ventricular dysfunction, with or without cardiac dilatation.^9^ Though the risk of potentially fatal myocarditis or cardiomyopathy with clozapine is estimated to be as low as 0.015%–0.188%,^10^ it is suggested that extreme caution should be exercised when using the drug, especially in the elderly.

## References

[1] Stephen S, Colin H, David O, Stoudemire A, Fogel BS, Greenberg DB (2000). Diagnosis and treatment of the dementias. Psychiatric care of the medical patient.

[2] Ung Gu Kang, Jun Soo Kwon, Yong Min Ahn (2000). Electro-cardiographic abnormalities in patients treated with clozapine. J Clin Psychiatry.

[3] Stuart JE, Jonathan EL, Tierney LM, Mcphee SJ, Papadakis MA (2004). Psychiatric disorders. Current medical diagnosis and treatment.

[4] Stephen MS (2005). Essential psychopharmacology: The prescriber's guide.

[5] Semple D, Smyth R, Burns J (2005). Clozapine. Oxford handbook of psychiatry.

[6] Henderson DC, Nguyen DD, Copeland PM (2005). Clozapine, diabetes mellitus, hyperlipidemia, and cardiovascular risks and mortality: Results of a 10-year naturalistic study. J Clin Psychiatry.

[7] Fineschi V, Neri M, Riezzo I (2004). Sudden cardiac death due to hypersensitivity myocarditis during clozapine treatment. Int J Legal Med.

[8] David BM, Susanne EA, Jean-Marie EB (2006). Myocarditis during clozapine treatment. Am J Psychiatry.

[9] Arthur MF, Dennis MN (2000). Myocarditis. N Engl J Med.

[10] Merrill DB, Dec GW, Goff DC (2005). Adverse cardiac effects associated with clozapine. J Clin Psychopharmacol.

